# *IDH1/2* mutations in acute myeloid leukemia patients and risk of coronary artery disease and cardiac dysfunction—a retrospective propensity score analysis

**DOI:** 10.1038/s41375-020-01043-x

**Published:** 2020-09-18

**Authors:** Badder Kattih, Amir Shirvani, Piroska Klement, Abel Martin Garrido, Razif Gabdoulline, Alessandro Liebich, Maximilian Brandes, Anuhar Chaturvedi, Timon Seeger, Felicitas Thol, Gudrun Göhring, Brigitte Schlegelberger, Robert Geffers, David John, Udo Bavendiek, Johann Bauersachs, Arnold Ganser, Joerg Heineke, Michael Heuser

**Affiliations:** 1 Department of Cardiology and Angiology, Hannover Medical School, Carl-Neuberg Strasse 1, 30625 Hannover, Germany; 2 Department of Cardiovascular Physiology, European Center for Angioscience (ECAS), Medical Faculty Mannheim of Heidelberg University, Ludolf-Krehl-Strasse 7-11, 68167 Mannheim, Germany; 3 Institute for Cardiovascular Regeneration, Goethe University Frankfurt, Theodor-Stern-Kai 7, 60590 Frankfurt am Main, Germany; 4 German Center for Cardiovascular Research (DZHK), partner site Rhein/Main, Frankfurt am Main, Germany; 5 Department of Hematology, Hemostasis, Oncology and Stem Cell Transplantation, Hannover Medical School, Carl-Neuberg Strasse 1, 30625 Hannover, Germany; 6 Department of Medicine III, University Hospital Heidelberg, Im Neuenheimer Feld 410, 69120 Heidelberg, Germany; 7 German Center for Cardiovascular Research (DZHK), partner site Heidelberg/Mannheim, Heidelberg, Germany; 8 Department of Human Genetics, Hannover Medical School, Hannover, Germany; 9 Genome Analytics, Helmholtz Center for Infection Research, Braunschweig, Germany

**Keywords:** Acute myeloid leukaemia, Acute myeloid leukaemia, Cancer genetics

## Abstract

Clonal hematopoiesis of indeterminate potential (CHIP) is linked to leukemia gene mutations and associates with an increased risk for coronary artery disease and poor prognosis in ischemic cardiomyopathy. Two recurrently mutated genes in CHIP and adult acute myeloid leukemia (AML) encode for isocitrate dehydrogenases 1 and 2 (*IDH1* and *IDH2*). Global expression of mutant *IDH2* in transgenic mice-induced dilated cardiomyopathy and muscular dystrophy. In this retrospective observational study, we investigated whether mutant *IDH1/2* predisposes to cardiovascular disease in AML patients. Among 363 AML patients, *IDH1* and *IDH2* mutations were detected in 26 (7.2%) and 39 patients (10.7%), respectively. Mutant *IDH1* patients exhibited a significantly higher prevalence of coronary artery disease (26.1% vs. 6.4%, *p* = 0.002). Applying inverse probability-weighting analysis, patients with *IDH1/2* mutations had a higher risk for a declining cardiac function during AML treatment compared to *IDH1/2* wild type patients [left ventricular ejection fraction pretreatment compared to 10 months after diagnosis: 59.2% to 41.9% (*p* < 0.001) vs 58.5% to 55.4% (*p* = 0.27), respectively]. Mechanistically, RNA sequencing and immunostaining in hiPS-derived cardiomyocytes indicated that the oncometabolite R-2HG exacerbated doxorubicin mediated cardiotoxicity. Evaluation of *IDH1/2* mutation status may therefore help identifying AML patients at risk for cardiovascular complications during cytotoxic treatment.

## Introduction

Acute myeloid leukemia (AML) is a clonal malignant disease of undifferentiated myeloid precursor cells, which develops due to somatically acquired driver mutations [1]. The isoforms 1 and 2 of *isocitrate dehydrogenase* (*IDH1* and *IDH2*) are two frequently mutated genes in adult AML. *IDH1* and *IDH2* encode enzymes involved in citrate metabolism that convert isocitrate to α-ketoglutarate (αKG), which is required for the biological activity of diverse dioxygenases (including TET2) [2, 3]. Mutant *IDH* converts αKG to R-2-hydroxyglutarate (R-2HG), a competitive inhibitor of αKG-dependent enzymes and a known oncometabolite [4, 5]. As *IDH1* and *IDH2* mutant tumors selectively produce R-2HG, oncometabolite measurements were found to provide useful diagnostic and prognostic information for IDH-targeted therapies [6–9]. Although several studies confirmed the high prevalence of *IDH* mutations in AML, the prognostic impact remains controversial with conflicting evidence [3, 10–12], because *IDH* mutations frequently co-occur with *NPM1* mutations, *MLL* partial tandem duplications, *FLT3*-ITD, or trisomy 8 [13–16]. Importantly, our group has recently shown that mutant *IDH1* and *IDH2* require additional oncogenes to induce leukemia in vivo and might also drive clonal expansion and evolution independent of its oncometabolite R-2HG [4, 17]. Interestingly, clonal hematopoiesis of indeterminate potential (CHIP) driven by leukemia-associated gene mutations has been associated with all-cause mortality due to an increased risk of coronary artery disease (CAD) [18, 19] and heart failure [20]. Recently, Mas-Peiro et al. showed that mutations in the most common CHIP driver genes *DNMT3A* and *TET2* may also be significantly associated with the progression of degenerative aortic valve stenosis [21]. Together, these data suggest that acquired somatic mutations in hematopoietic cells may represent an important risk factor for the progression of cardiovascular disease. Therefore, the purpose of this study was (1) to determine whether an acquired driver mutation in *IDH1 or IDH2* predisposes AML patients toward cardiac dysfunction or even triggers myocardial susceptibility to antineoplastic treatments with cardiotoxic agents (such as anthracyclines), and (2) to integrate the novel findings from this translational retrospective study into the clinical context of cardio-oncology as a novel and rapidly growing research area.

## Results

### Patient characteristics, detection of *IDH1*/*2* mutations, and prognostic significance

We analyzed a total of 363 patients with AML, whereby 298 (82.1%) had no *IDH1/2* mutation (*IDH1/2*^wildtype^AML), and 65 (17.9%) exhibited either an *IDH1* or an *IDH2* mutation. Among all 363 tested AML patients, an *IDH1* mutation was detected in 26 (7.2%) cases and an *IDH2* mutation in 39 cases (10.7%). The baseline characteristics of both groups (*IDH1/2*^wildtype^AML vs. *IDH1*^mutated^AML and/or *IDH2*^mutated^AML) are summarized in Table 1. The median age of the total population was 60 years. Secondary and therapy-related AML were diagnosed in 25.1% and 6.1 % of the patients, respectively. Favorable, intermediate, or adverse cytogenetic risk was found in 21.6%, 54.7%, and 23.7% of all patients, respectively. Intensive induction chemotherapy was administered in 82.2% of the patients. Of these, 37.8% were consolidated with chemotherapy and 62.2% received an allogeneic transplant in first complete remission (CR). Patient characteristics were similar between *IDH1*^mutated^ or *IDH2*^mutated^ patients and *IDH1/2*^wildtype^ patients except for a higher platelet count (*p* = 0.019) as well as a more frequent diagnosis of antecedent CMML (*p* = 0.035) in *IDH1/2* mutated compared to *IDH1/2*^wildtype^ patients (Table 1). In the subgroup of patients treated with intensive induction chemotherapy, 196 (66.4%) patients achieved CR with similar CR rates between *IDH1/2*^wildtype^, *IDH1*^mutated^, and *IDH2*^mutated^ patients (65.7%, 63.6%, 74.2%, *p* = 0.615) (Table S1 in the Data Supplement). Importantly, the cumulative dose of anthracyclines did not differ according to mutational status and was comparable in all comparator groups (IDH^WT^ vs. IDH^mut^) (Table 1).

**Table 1:** Baseline characteristics of the AML study cohort (*IDH*^wildtype^ vs. *IDH1*^mutated^ or *IDH2*^mutated^ vs. pooled *IDH1*^mutated^ and *IDH2*^mutated^).

	Unweighted cohort (total *n* = 363)											
	AML total		*IDH* wildtype		*IDH1* mutated		*IDH2* mutated			*IDH1/2* mutated		
	(Total *n* = 363)	a.d. (*n*/total *n*)	(Total *n* = 298)	a.d. (*n*/total *n*)	(Total *n* = 26)	a.d. (*n*/total *n*)	(Total *n* = 39)	a.d. (*n*/total *n*)	*p*	(Total *n* = 65)	a.d. (*n*/total *n*)	*p*
Age (years)		363/363		298/298		26/26		39/39	0.389		65/65	0.242
Median	60.0		60.0		62.0		62.0			62.0		
Range	18–90.0		18.0–90.0		31.0–82.0		27.0–81.0			27.0 – 82.0		
Age ≤60 years (%)	50.1	182/363	50.7	151/298	46.2	12/26	48.7	19/39	0.891	47.7	31/65	0.663
Age >60 years (%)	49.9	181/363	49.3	147/298	53.8	14/26	51.3	20/39		52.3	34/65	
Male sex (%)	57.6	209/363	56.7	169/298	69.2	18/26	56.4	22/39	0.459	61.5	40/65	0.476
AML history (%)		363/363		298/298		26/26		39/39	0.592		65/65	0.501
De novo AML	68.9	250/363	68.8	205/298	76.9	20/26	64.1	25/39		69.2	45/65	
Secondary AML	25.1	91/363	24.5	73/298	23.1	6/26	30.8	12/39		27.7	18/65	
Therapy-related AML	6.1	22/363	6.7	20/298	0.0	0/26	5.1	2/39		3.1	2/65	
WBC at diagnosis (/µl)		356/363		292/298		26/26		38/39	0.733		64/65	0.464
Median	8.6		8.6		6.9		11.6			8.8		
Range	0.3–284.0		0.3–284.0		0.7–206.1		0.9–146.3			0.7–206.1		
Platelet count at diagnosis (/µl)		293/363		241/298		22/26		30/39	0.064		52/65	0.019
Median	55.0		50.0		83.5		94			85.5		
Range	2.0–979.0		2.0–523.0		10.0–469.0		7.0–979.0			7.0–979.0		
Hemoglobin (g/dl)		290/363		237/298		22/26		31/39	0.528		53/65	0.404
Median	9.2		9.1		9.6		9.3			9.6		
Range	2.5–16.6		2.5–16.0		4.9–12.1		5.5–16.6			4.9–16.6		
Blood blasts (%)		155/363		130/298		12/26		13/39	0.348		25/65	0.166
Median	38.3		33.0		55.5		46.7			46.7		
Range	0.0–98.0		0.0–98.0		4.0–89.0		0.0–90.0			0.0–90.0		
Bone marrow blasts (%)		131/363		108/298		17/26		15/39	0.421		23/65	0.256
Median	60.0		60.0		85.0		70.0			80.0		
Range	0.0–100.0		0.0–99.0		20.0–96.0		8.0–100.0			8.0–100.0		
Cytogenetic risk group (%)		342/363		279/298		25/26		38/39	0.436		63/65	0.403
Favorable	21.6	74/342	22.2	62/279	28.0	7/25	13.2	5/38		18.5	12/65	
Intermediate	54.7	187/342	55.6	155/279	44.0	11/25	55.3	21/38		49.2	32/65	
Adverse	23.7	81/342	22.2	62/279	28.0	7/25	31.6	12/38		29.2	19/65	
NPM1 mutation (%)	16.8	61/363	17.4	52/298	18.2	4/22	14.7	5/34	0.807	13.8	9/65	0.570
FLT3-ITD presence (%)	15.4	56/363	15.8	47/298	22.7	5/22	11.8	4/34	0.551	13.8	9/65	0.792
ECOG performance status (%)		358/358		294/294		26/26		38/38	0.656		68/68	0.809
ECOG 0-1	65.4	234/358	65.6	193/294	57.7	15/26	68.4	26/38		64.1	41/64	
ECOG 2-4	34.6	124/358	34.4	101/294	42.3	11/26	31.6	12/38		35.9	23/64	
Antecedent CMML (%)	2.5	9/363	1.7	5/298	3.8	1/26	7.7	3/39	0.068	6.2	4/65	0.035
Intensive induction therapy (%)	82.2	295/359	82.3	242/294	84.6	22/26	79.5	31/39	0.860	81.5	53/65	0.883
Anthracyclines (mg/m^2^**)**												
Median	240.0	295/359	240.0	242/294	240.0	22/26	192.0	31/39	0.242	192.0	53/65	0.186
Range	0.0–360.0		0.0–360.0		60.0–360.0		144.0–360.0			60.0–360.0		
Consolidation type (%)		312/358		252/298		24/26		36/36			60/60	
Chemotherapy	37.8	118/312	36.1	91/252	45.8	11/24	44.4	16/36	0.440	45.0	27/60	0.202
alloHCT	62.2	194/312	63.9	161/252	54.2	13/24	55.6	20/36		55.0	33/60	

Values are expressed as median and range or % (*n*/total *n*).

*a.d.* available data, *alloHCT* allogeneic hematopoietic cell transplantation, *CMML*  chronic myelomonocytic leukemia, *ECOG* Eastern Co-operative Oncology Group, *FLT3-ITD* FMS-like tyrosine kinase 3 internal tandem duplication, *NPM1* Nucleophosmin 1.

**Fig. 1:**
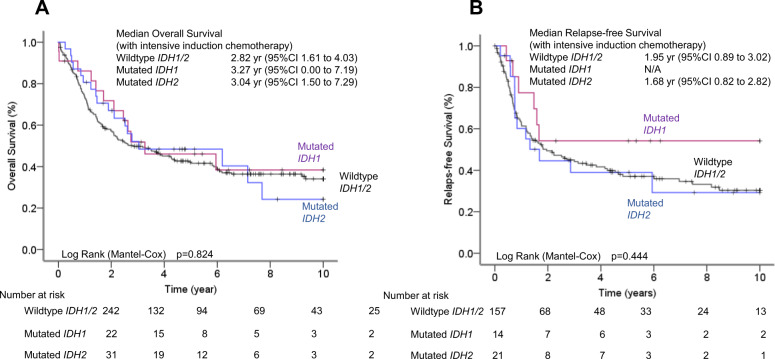
Outcome of adult AML patients according to induction chemotherapy status. **a** Overall survival and **b** Relapse-free survival in AML patients treated with intensive induction and consolidation chemotherapy according to *IDH1* and *IDH2* mutation status in patients with mutated *IDH1* and *IDH2*.

Overall survival (OS) and relapse free survival (RFS) were investigated in the subgroup of 295 patients who were treated with intensive induction chemotherapy. During a median follow-up of 7.6 years (95% confidence interval, 6.9–8.2 years), the estimated 2-year RFS and OS rates in the study cohort were 49.4% (5-year RFS 38.9%) and 59.2% (5-year OS 43.1%), respectively. RFS and OS in the study cohort did not significantly differ between *IDH1/2*^wildtype^ and *IDH1*^mutated^ or *IDH2*^mutated^ or pooled *IDH1/2*^mutated^ AML patients (Fig. 1 and Table S2 in the Data Supplement).

### Association between *IDH* mutation status and cardiovascular baseline characteristics

**Fig. 2:**
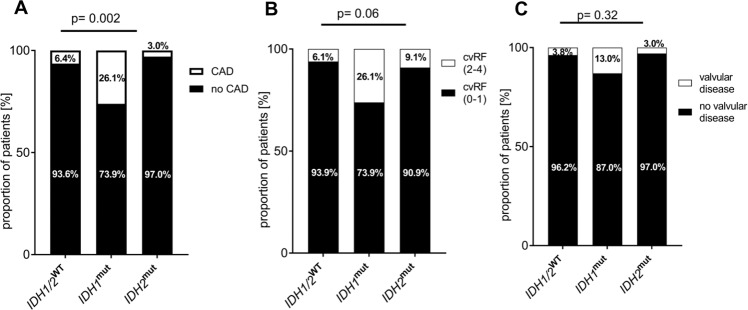
Association between *IDH* mutation status in AML and the frequency of  cardiovascular disease. **a** Coronary artery disease (CAD), **b** cardiovascular risk factors (cvRF) and **c** valvular disease.

We next examined whether the *IDH* mutation status in AML patients is associated with cardiovascular disease. AML patients with *IDH1* mutation more often had a history of CAD compared to *IDH1/2*^wildtype^ patients (26.1% vs. 6.4%; *p* = 0.002) and tended to present with a higher percentage of cardiovascular risk factors (26.1% vs. 10.1% *p* = 0.06) and valvular disease (13.0% vs. 3.8%, *p* = 0.32) than AML patients with *IDH1/2*^wildtype^ (Fig. 2 and Table 2). Other cardiovascular baseline characteristics were similar between *IDH1/2*^wildtype^ versus *IDH1*^mutated^ AML patients including baseline heart rate, blood pressure and heart failure medication (Table 2). Cardiovascular baseline characteristics did not differ between *IDH2*^mutated^ and *IDH1/2*^wildtype^ nor between pooled *IDH1/2*^mutated^ and *IDH1/2*^wildtype^ (Table 2).

**Table 2:** Cardiovascular characteristics of the AML study cohort (*IDH*^wildtype^ vs. *IDH1*^mutated^ or *IDH2*^mutated^ vs. pooled *IDH1*^mutated^ and *IDH2*^mutated^).

	Unweighted cohort (total *n* = 363)											
	AML total		*IDH* wildtype		*IDH1* mutated		*IDH2* mutated			*IDH1/2* mutated		
	(Total *n* = 363)	a.d. (*n*/total *n*)	(Total *n*= 298)	a.d. (*n*/total *n*)	(Total *n* = 26)	a.d. (*n*/ total *n*)	(Total *n* = 39)	a.d. (*n*/total *n*)	*p*	(Total *n* = 65)	a.d. (*n*/total *n*)	*p*
CAD (%)	7.5	(24/321)	6.4	(17/265)	26.1	(6/23)	3.0	(1/33)	0.002	12.5	(7/56)	0.116
Valvular disease (%)	4.4	(14/321)	3.8	(10/265)	13.0	(3/23)	3.0	(1/33)	0.323	7.1	(4/56)	0.403
Heart failure (%)	2.5	(8/320)	2.3	(6/264)	0.0	(0/23)	6.1	(2/33)	0.307	3.6	(2/56)	0.572
Cardiovascular risk factors (%)									0.060			0.198
0–1 risk factors	88.9	(287/323)	89.9	(240/267)	73.9	(17/23)	90.9	(30/33)		83.9	(47/56)	
2–4 risk factors	11.1	(36/323)	10.1	(27/267)	26.1	(6/23)	9.1	(3/33)		16.1	(9/56)	
Sinusrhythm (%)	91.8	(292/318)	92.0	(242/263)	90.9	(20/22)	90.9	(30/33)	0.964	90.9	(50/55)	0.785
Systolic blood pressure (mmHg)	35.9	(127/354)		(100/289)		(11/26)		(16/39)	0.268		(27/65)	0.282
Median			130.0		120.0		130.0			125.0		
Range			80.0–180.0		110.0–140.0		110.0–150.0			110.0–150.0		
Diastolic blood pressure (mmHg)	35.3	(125/354)		(98/289)		(11/26)		(16/39)	0.884		(27/65)	0.791
Median			75.0		80.0		80.0			80.0		
Range			40.0–105.0		60.0–90.0		65.0–90.0			60.0–90.0		
Heart rate (beats per min)	49.9	(181/363)		(150/298)		(14/26)		(17/39)	0.427		(31/65)	0.825
Median			88.0		93.5		84.0			88.0		
Range			60.0–152.0		62.0–122.0		66.0–118.0			62.0–120.0		
ACEi/ARB (%)	18.9	(60/317)	17.6	(46/262)	26.1	(6/23)	25.0	(8/32)	0.395	25.5	(14/55)	0.174
ß-blocker (%)	17.9	(57/317)	16.8	(44/262)	30.4	(7/23)	18.8	(6/32)	0.262	23.6	(13/55)	0.230
MR-antagonists (%)	0.0	(0/317)	0.8	(2/262)	0.0	(0/23)	0.0	(0/32)	0.810	0.0	(0/55)	0.516

Values are expressed as median and range or % (*n*/total *n*).

*ACEi* angiotensin converting enzyme inhibitor, *ARB* angiotensin receptor blocker, *CAD* coronary artery disease, *MR-antagonists* mineralocorticoid-receptor antagonists, *a.d.* available data.

### Association between *IDH* mutation status and cardiac function

To assess whether the *IDH* mutation status in AML patients has an impact on cardiac function, we analyzed left ventricular ejection fraction (LVEF) by echocardiography in the control group (AML patients without *IDH* mutation) and the exposed group (AML patients with *IDH1/2* mutation) before and at different time points during or after AML therapy.

Because potential confounding factors for the outcome parameter (LVEF) were unequally distributed (with regard to covariate balance assessed by the standardized mean differences), the inverse probability weighting (IPW) method was used to create a weighted study sample, in which the distribution of potential confounding factors is independent of *IDH* mutation status. The IPW method based on the propensity score is an established statistical technique to minimize bias in observational studies by weighting individual samples with similar characteristics for those subjects of the study population which were under-represented or should have been included in the study to achieve covariate balance (thereby simulating additional observations without being affected by selection bias) [22–24]. We chose this approach to evaluate whether mutations of the *IDH* genes in AML patients versus the control group affect cardiac function before and during AML therapy (measured as LVEF by echocardiography in the available subset of patients). As *IDH1*^mutated^ AML patient characteristics significantly associated with cardiovascular disease as a potentially independent risk factor, we primarily balanced prognostically important baseline covariates to compare *IDH1/2*^wildtype^ with *IDH2*^mutated^ AML patients and *IDH1/2*^wildtype^ with the pooled *IDH1*^mutated^ and *IDH2*^mutated^ cohort. Therefore, two separate IPW models were employed to allow direct comparisons of *IDH1/2*^wildtype^ with *IDH2*^mutated^ AML patients (model 1) as well as *IDH1/2*^wildtype^ with the pooled *IDH1*^mutated^ and *IDH2*^mutated^ cohorts (model 2).

**Fig. 3:**
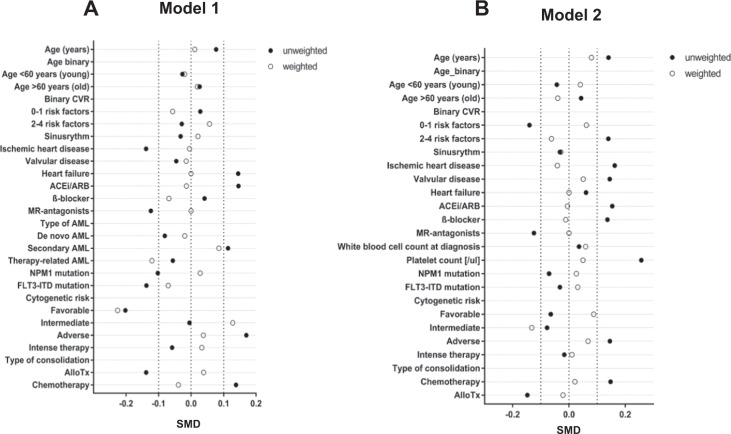
Comparison of covariate balance in the  weighted and unweighted study cohort by using absolute standardized differences. **a** In model 1 between *IDH2*^mutated^ (exposed) and *IDH1/2*^wildtype^ (non-exposed) or **b** in model 2 between pooled *IDH1/2*^mutated^ and *IDH1/2*^wildtype^ in the unmatched (white circles) and the matched study sample (black circles). The standardized difference is the difference of the mean values or proportions (exposed–non-exposed group) divided by the pooled standard deviation. It measures the effect size between two groups and is independent from sample sizes. Inverse probability of treatment weighting has reduced many of the systematic differences between exposed and non-exposed subjects and resulted in balance in the measured variables. Raw values are shown in Supplementary Tables S3 and S4.

**Fig. 4:**
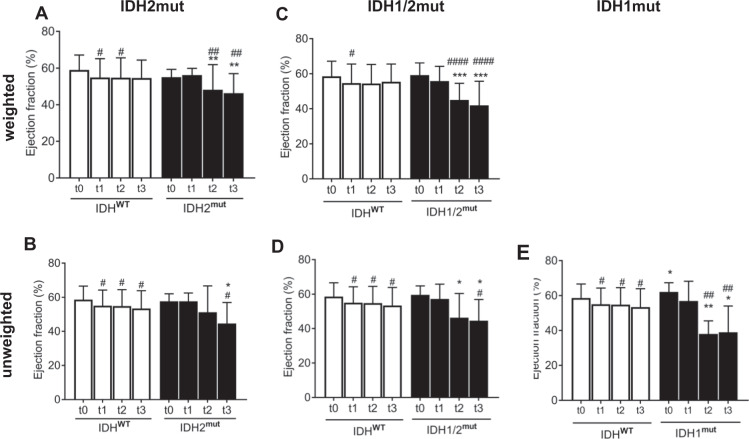
Course of left ventricular ejection fraction (measured by echocardiography) in patients suffering from AML. **a** Ejection fraction in the weighted study cohort comparing *IDH2* versus wildtype *IDH1/2* and **b** in the unweighted study cohort. **c** Left ventricular ejection fraction in the weighted study cohort comparing *IDH1/2* mutated versus wildtype *IDH1/2* **d** and in the unweighted study cohort. **e** Ejection fraction in the unweighted study cohort comparing *IDH1* mutated versus wildtype *IDH1/2*. *p < 0.05, **p < 0.01, ***p < 0.001 comparing wildtype *IDH1/2* with mutated *IDH2* at the corresponding timepoints (*t*_x_). #*p* < 0.05, ##*p* < 0.01, ###*p* < 0.001 comparing the corresponding timepoint within the same group with timepoint *t*_0_. *t*_0_ denotes the timepoint prior to AML therapy and *t*_1–3_ show the ejection fraction at different timepoints after AML therapy. Mut mutated, WT wildtype. All data shown are mean, error bars represent ±SD.

Weighting the study populations using the IPW method resulted in an overall balance of baseline covariates and standardized mean differences (except for favorable cytogenetic risk and type of AML in model 1 and cytogenetic risk in model 2), indicating that the weighted study cohorts in both models were comparable with regard to measured covariates (Fig. 3 and Supplementary Tables S3, S4 in the Data Supplement). Clinical outcomes of the weighted and unweighted cohorts are shown in Fig. 4 and Supplementary Tables S5–S7 in the Data Supplement. In model 1 *IDH2*^mutated^ patients cardiac function declined during AML therapy (*t*_2_ and *t*_3,_ e.g,. *t*_3_: absolute LVEF reduction −8.3%, CI −15.88 to −0.72, *p* = 0.005) compared to *IDH1/2*^wildtype^AML patients (Fig. 4a and Supplementary Table S5 in the Data Supplement), indicating that the *IDH2* mutation status in AML patients is associated with impaired left ventricular ejection fraction during AML therapy, which is independent of measured baseline covariates. In the corresponding unweighted study cohort (*IDH1/2*^wildtype^ versus *IDH2*^mutated^), cardiac function declined without differences between *IDH1/2*^wildtype^ and *IDH2*^mutated^ patients at earlier time points (*t*_1_–*t*_2_) during AML therapy, while at the latest time point t_3_ *IDH2*^mutated^ patients exhibited a significantly reduced LVEF (*t*_3:_ absolute LVEF reduction −8.8%, 95%CI −18.78 to 1.18, *p* = 0.028) compared to *IDH1/2*^wildtype^ patients (Fig. 4b and Supplementary Table S5 in the Data Supplement).

When comparing *IDH1/2*^wildtype^ and pooled *IDH1/2*^mutated^ AML patients in the weighted study cohort (model 2), the pooled *IDH1/2*^mutated^ group showed a reduced LVEF after the initiation of AML therapy (in *t*_2_ and *t*_3_; e.g., *t*_3_: absolute LVEF reduction −13.5%, 95%CI −20.94 to −6.06, *p* < 0.0001) compared to *IDH1/2*^wildtype^ AML patients (Fig. 4c and Supplementary Table S6 in the Data Supplement), suggesting a possible link between mutations in the *IDH1/2* genes of hematopoietic cells in AML and an impaired LVEF following initiation of AML therapy after achieving covariate balance. In the corresponding unweighted study cohort, the mean LVEF declined at different time points during AML therapy in both groups with significantly reduced ejection fraction in *IDH1/2*^mutated^ AML compared to *IDH1/2*^wildtype^ patients after the initiation of AML therapy (*t*_2–3_, e.g., *t*_3_: absolute LVEF reduction −8.8%, 95%CI −18.80 to 1.20, *p* = 0.028) (Fig. 4d and Supplementary Table S6 in the Data Supplement).

When comparing (*IDH1/2*^wildtype^ versus *IDH1*^mutated^) in the unweighted study cohort, cardiac function declined at different time points following AML therapy in *IDH1/2*^wildtype^ and *IDH1*^mutated^ patients, while *IDH1* mutation was significantly related with deterioration of cardiac function following the initiation of AML therapy (*t*_2–3_, e.g., *t*_2_: absolute LVEF reduction −16.8%, 95%CI −27.59 to −6.01, *p* = 0.002) (Fig. 4e and Supplementary Table S7 in the Data Supplement).

### Oncometabolite R-2HG impairs sarcomere organization and exacerbates cardiotoxicity of doxorubicin in hiPS-derived cardiomyocytes (CMs)

**Fig. 5:**
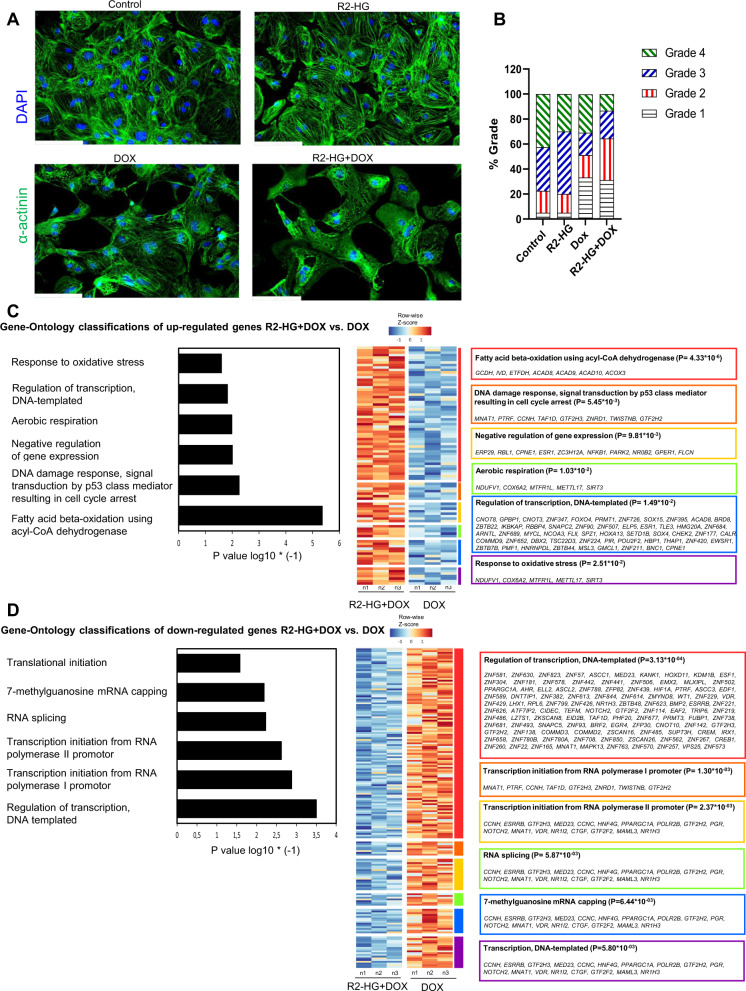
The oncometabolite R-2HG exacerbates the maladaptive effects of doxorubicin in human iPS-derived cardiomyocytes. Representative images for sarcomere organization in human iPS-derived CMs immunostained for α-actinin and exposed to doxorubicin 1 µM and/ or R-2HG 20 mM (**a**) assessed using a semiquantitative grading system (Scale bar 100 µm). (**b**) Gene-Ontology (Biological Process) analysis showing the functional categories and the identity of enriched upregulated (**c**) or downregulated genes (**d**) with corresponding heat maps after R-2HG exposure in doxorubicin treated human iPS-derived CMs from the RNA sequencing experiment.

Because *IDH1/2* mutations in patients with AML were associated with a pronounced decline in cardiac function during AML therapy, we hypothesized that these effects (at least in part) might be attributed to a detrimental effect of the oncometabolite R-2HG) on cardiomyocytes. To validate whether R-2HG increases myocardial vulnerability to anthracyclines, we exposed human induced pluripotent stem cell (hiPS)-derived CMs to 1 µM doxorubicin (DOX) with or without the addition of R2-HG. Immunostaining analysis using a semiquantitative grading system revealed a decreased sarcomere organization in hiPS-derived CMs exposed to DOX compared to untreated control CMs (Fig. 5a). While R2-HG alone did not affect sarcomere organization, co-incubation of R2-HG and DOX further aggravated the loss of sarcomere organization in hiPS-derived CMs (e.g., 33.3% vs 17.9%, grade 2) compared to DOX alone (Fig. 5b), suggesting an increased structural vulnerability of R-2HG exposed myocardial cells during anthracycline containing chemotherapy.

In addition, we isolated RNA from hiPS-derived CMs and performed RNA-sequencing (RNAseq) to decipher differential regulation of the transcriptome that might contribute to the observed cardiac phenotype in mutant *IDH* AML patients. In DOX treated hiPS-derived CMs (compared to control), 675 genes were downregulated, and enriched genes were related to the GO (biological process) classes chromatid cohesion, cell division, mitochondrial translational elongation, DNA repair, initiation of DNA replication, or extrinsic apoptotic signaling pathways (Supplementary Fig. S1a). In addition, 904 genes were upregulated, and enriched genes were linked to GO classes negative regulation of cell proliferation, nucleosome assembly, epithelial to mesenchymal transition, or positive regulation of apoptotic processes (Supplementary Fig. S1b).

However, our aim was to identify the R-2HG dependent biological processes that exacerbated cardiotoxicity during DOX treatment. Therefore, we also compared the mRNA expression pattern between R-2HG+ DOX and DOX treatment alone. By analysis of Gene-ontology terms (biological process), we identified the upregulation of 519 genes, and enriched genes were involved in biological processes like fatty acid beta-oxidation, DNA damage response or response to oxidative stress genes (Fig. 5c). Importantly, we observed the downregulation of a large number of genes (1028) involved in crucial cell housekeeping functions such as transcription (both from polymerase I and II promotors), RNA splicing and capping as well as translational initiation. Genes with significant differential regulation between both conditions were categorized by GO classes and are shown in the heatmap (Fig. 5d). Our data suggest that the oncometabolite R-2HG contributes, at least in part, to the increased cardiotoxicity during anthracycline treatment observed in this study.

## Discussion

In this retrospective study we found that *IDH1/2* mutations in patients with AML are associated with a more rapid decline of LVEF during AML therapy. Although we cannot infer causality from this analysis, the average treatment effect revealed that patients with *IDH* mutations in AML displayed significantly reduced LVEF during AML therapy after achieving a balanced distribution of baseline characteristics between the *IDH1/2*^wildtype^ and *IDH1/2*^mutated^ groups based on inverse probability of treatment weighting. In addition, an *IDH1* mutation in AML was associated with a higher percentage of CAD at diagnosis of AML in an unweighted analysis. *IDH1/2* mutations did not affect relapse-free survival or crude overall survival in this study cohort. In addition, the decrease in LVEF during treatment was stronger in *IDH2* as well as *IDH1* mutated patients and the combined group of *IDH1/2* mutated patients compared to *IDH1/2* wildtype patients, suggesting an increased vulnerability of R-2HG exposed myocardial cells during cytarabine/anthracycline containing chemotherapy. Indeed, we further demonstrate that the decline in cardiac function in AML patients with mutant *IDH* following anthracycline therapy might be attributed to an effect of oncometabolite R-2HG. While R-2HG alone had no effect, it enhanced the doxorubicin mediated sarcomere disarray in hiPS-derived CMs. By RNA sequencing, we show the transcriptomic basis for putative biological processes driving the increased cardiotoxicity of R2-HG exposed cardiomyocytes during doxorubicin treatment. While previous studies have pointed toward a significant association of CHIP mutations (like *DNMT3A* and *TET2*) with the risk of CAD [18, 19], progression of degenerative aortic valve stenosis [21], and impaired prognosis in patients with heart failure [20], this study is the first to suggest that *IDH1* mutations are also associated with CAD and cardiac dysfunction in established AMLs. Experimental studies confirmed that genetic TET2 deficiency in murine hematopoietic stem cells promotes cardiac dysfunction and accelerates atherosclerosis in mice [25–27]. Notably, *IDH1* and *IDH2* mutations also occur as CHIP mutations [28] and mechanistically lead to conversion of αKG to R-2HG, which is a proven oncometabolite and a potent inhibitor of TET2 [4, 29], suggesting a common pathway of CHIP induced cardiac disease. Alternatively, preclinical and genetic studies have demonstrated that increased amounts of the oncometabolite R-2HG, produced by *IDH* mutant leukemic cells, cause cardiac dysfunction by impairing cellular metabolism in the heart [30]. Moreover, transgenic mice with global expression of mutant *IDH2* develop dilated cardiomyopathy accompanied with elevated R-2HG levels, while in turn, silencing of mutant *IDH2* expression restored cardiac function by lowering R-2HG levels [31]. Together, these mechanistic studies suggest that *IDH* mutant cells might directly or indirectly predispose to cardiac deterioration in patients. Although our clinical study was not designed to provide the entirety of the molecular mechanisms that contribute to the worsening of cardiac function by mutated *IDH*, a global transcriptome analysis by RNA sequencing of R2-HG exposed hiPS CMs under doxorubicin treatment revealed a distinct downregulation of a high number of genes involved in basic cellular housekeeping functions that will aggravate anthracycline toxicity. This gene-regulation pattern might affect sarcomere assembly in cardiomyocytes, which highly depend on intact transcriptional and translational processes [32]. Although sarcomeric disarray is also a well-known effect of doxorubicin on cardiomyocytes [33–35], we further demonstrate that R-2HG potentiated the doxorubicin mediated deterioration of sarcomere organization in hiPS CMs. Notably, R-2HG exposure during doxorubicin treatment (compared to doxorubicin alone) induced further upregulation of detrimental biological processes (like response to oxidative stress or DNA damage response) that are well-established effects of doxorubicin, suggesting an exacerbated cardiotoxicity.

In light of this preclinical evidence, our results provide support that *IDH* mutations in clonal hematopoiesis may play an essential role as a cardiovascular risk factor in driving cardiovascular disease progression, which should be taken into account especially prior to initiation of chemotherapy, because left ventricular dysfunction is a common side effect of cancer treatment [36]. Anthracyclines, for example, are regularly used for AML treatment and have known cardiotoxic effects in a dose-dependent manner in up to 10% of patients. [3, 36–38] In this study the stronger decrease in LVEF (up to −8.3% to −16.8%) in *IDH* mutated AML patients during treatment might be of clinical importance since in line with the definition of cardiotoxicity commonly used, cancer treatment-related cardiac dysfunction is relevant with a decrease in the LVEF of 10 percentage points and these patients have a better cardiac outcome when treated with ACE inhibitors and/or beta-blockers early after detection of cardiac dysfunction [36, 39, 40]. Therefore, our study might provide a basis for future clinical risk assessment, since patients with anthracycline-associated cardiac dysfunction frequently have the potential to exert functional myocardial recovery (provided that it is detected early and treated with heart failure medications). Conversely, late identification of LV dysfunction is associated with impaired response to medication [36, 39, 41]. Thus, careful baseline assessment of cardiovascular risk factors and genetic risk factors including the *IDH* mutation status as well as baseline measurement of cardiac function by a cardio-oncology specialist team should be performed in these AML patients (especially when adjuvant anthracyclines are used). It will be interesting whether *IDH* inhibitors, that effectively reduce R-2HG levels in vivo, can reduce the cardiovascular risk observed in our study [42–46].

Despite the relatively small number of patients, our AML cohort appears representative of the general AML population, since the overall frequency of *IDH* mutations among AML patients in this study was 17.9% (with an *IDH1* frequency of 7.2% and an *IDH2* frequency of 10.7%), which is similar to those reported by others [15, 47]. Accordingly, in our study cohort *IDH* mutant patients also tended to be older, to have higher platelet counts and percentages of blood or bone marrow blasts, which was also reported by previous studies [10–12, 15, 47] with individual differences due to the size or varying inclusion criteria of the studies. In accordance with previous studies [12, 15, 48–51] and a recent meta-analysis by Xu et al. [52], *IDH* mutations had no impact on crude overall survival and relapse-free survival in our study cohort. Despite these points, some limitations of our study need to be emphasized: First, this is a “real-world” observational, retrospective, single center study with a limited number of AML patients harboring *IDH* mutations. Further studies will be needed to investigate the cardiovascular effects of *IDH* mutations in larger AML cohorts. Second, we had no information about oncometabolite R-2HG levels, which could have been correlated with prognostic outcome parameters and ejection fraction. Hence, we cannot directly correlate cardiovascular risk and cardiac dysfunction with R-2HG levels or with anthracycline exposure, although there should be little differences between the included patients regarding the latter because of the standardized AML treatments in this study cohort. Third, further studies are needed to investigate the effects of *IDH* mutations on cardiac dysfunction by more sensitive tools than echocardiographic ejection fraction to detect also subclinical myocardial dysfunction (e.g., by global longitudinal strain analysis, or MRI based measurements). Fourth, as with all retrospective observational studies due to their non-randomized nature, unmeasured confounders, and missing values may have affected our findings. In addition, we had no information about the presence of CHIP before the onset of AML in this study cohort. In order to ameliorate some of these limitations, a propensity score analysis was employed to balance differences in baseline characteristics between the two comparator groups in both models (accounting for age, cardiovascular risk profile, and heart failure medications). In this regard, it is remarkable that cardiac deterioration was detected while the study populations (after inverse probability of treatment weighting) exerted a similar cardiovascular risk profile and equally received heart failure medications.

In conclusion, mutant *IDH* in patients with AML was associated with increased risk for cardiac dysfunction and a higher prevalence of CAD at the time of AML diagnosis and during treatment with intensive chemotherapy. The impact of cardiovascular monitoring and optimal treatment of cardiovascular risk factors in these AML patients on cardiovascular outcomes should be further evaluated.

## Methods

### Study design and setting

This retrospective observational single-center study was conducted at Hannover Medical School. The data of in- and out-patients were obtained by using the medical administrative database for patient documentation. We investigated whether *IDH1* or *IDH2* mutations in patients with AML might have a prognostic impact and predispose to cardiac deterioration before, during or after AML therapy.

### Patient population and data collection

All patients 18 years or older with a diagnosis of AML and available peripheral blood or bone marrow sample at time of diagnosis being treated between 1996 and 2015 at Hannover Medical School were included in this study (excluding acute promyelocytic leukemia). The majority of patients were treated with intensive chemotherapy regimens within trials (SHG-0295 [53], SHG-0199 [53], AMLSG-0704 [54], AMLSG-0909 [55], or AMLSG-16-10 [56]) based on cytarabine/anthracycline induction chemotherapy and high-dose cytarabine or allogeneic hematopoietic cell transplantation (alloHCT) consolidation. The remaining patients were treated with non-intensive regimens including low-dose cytarabine, decitabine, azacitidine, hydroxyurea, or best supportive care. Collected data included demographics, cardiovascular risk factors, cardiac assessments as well as clinical characteristics, vital signs including systolic and diastolic blood pressure, heart rate, prescriptions, and several laboratory tests. Outcome parameter was LVEF assessed by cardiac imaging and was collected as part of routine diagnostics by different examiners. Notably, the values of echocardiographic ejection fraction were only available for a subset of patients. The results were calculated as group or cohort mean and tested with an unpaired *t*-test.

Written informed consent was obtained according to the Declaration of Helsinki, and the study was approved by the institutional review board of Hannover Medical School (3724-2004 and 936-2011). Timepoints (*t*_0–3_) are calculated as the median and mean time (month) from date of diagnosis to the date of echocardiography (Supplementary Table S8). Timepoint *t*_0_ denotes the timepoint before AML therapy and *t*_1–3_ show the ejection fraction at different timepoints during or after AML therapy (timepoints are shown in Supplementary Tables S5–8).

### Cytogenetic and molecular genetic analyses

Cytogenetic analyses were carried out using standard chromosome-banding analysis and using reverse transcription (RT)-PCR and/or fluorescent in situ hybridization for recurrent genetic abnormalities. Preparation of mononuclear cells and extraction of genomic DNA were performed as reported previously [57]. The genomic regions of exon 4 of the *IDH1/2* genes were analyzed in two sets using primers and PCR conditions as described [58, 59]. Purified PCR fragments were directly sequenced.

### Propensity score method

Due to the non-randomized nature of a retrospective observational analysis, a propensity score method was applied to yield a balanced distribution of baseline characteristics and to estimate the unbiased effects of *IDH* mutation status in AML patients on a cardiovascular outcome parameter. Briefly, for the final study population a propensity score was calculated using a logistic regression model, in which the *IDH* mutation status (model1: *IDH*^wildtype^ or *IDH2*^mutated^; model2: *IDH*^wildtype^ or pooled *IDH1*^mutated^, and *IDH2*^mutated^) was regressed as dependent (and binary) variable on relevant baseline characteristics (including cardiovascular risk factors, heart failure medication, cardiovascular disease) [60]. Since *IDH1*^mutated^ AML patient characteristics differed essentially from the other groups, which is one major finding of this study, we dispensed of adjusting a third model for *IDH1*^mutated^ AML versus *IDH1/2*^wildtype^. Variables included in the propensity score model 1 and 2 to achieve covariate balance are listed in Fig. 3 and Supplementary Tables S3, S4 in the Data Supplement. To balance prognostically important baseline covariates in both groups, the inverse probability of treatment weighting method was used to create a weighted study sample, in which the distribution of potential confounding factors is independent of *IDH* mutation status, allowing an unbiased estimate of the relationship between *IDH* mutation status and cardiac function with covariate balance between the exposed group (AML patients with *IDH* mutation) and the control group (AML patients without *IDH* mutation) (Fig. 3). Corresponding weights for patients in the AML cohort with mutated *IDH* were calculated by 1/PS and for those in the control cohort(AML without *IDH* mutation) by 1/(1-PS) [61]. To improve comparability between both groups (exposed and non-exposed) and to avoid residual confounding (due to extreme weights), the dataset was pre-processed by a typical trimming method thereby removing the most extreme 5% propensity scores [22]. Finally, a weight truncation method was used to reduce residual extreme weights (defined as weights >10) down to a threshold with a maximum weight of 10 [22]. The weights are incorporated in subsequent analyses comparing the cardiovascular outcome parameter (left ventricular ejection fraction) between both AML groups. Absolute standardized difference ≤0.1 for measured covariates suggested appropriate balance between the groups (Fig. 3 and Supplementary Tables S3, S4 in the Data Supplement). We chose a propensity score based method, which retains the patient data and creates a pseudo population with an optimal covariate balance, over multivariate analysis to better adjust for confounders in small datasets of nonrandomized studies, which appears less biased, more robust, and more precise than standard multivariable methods [62]. In addition, the propensity score method was preferred over a conventional multivariable analysis, as multicollinearity was a concern in the set of independent variables. Propensity score based methods allow estimating the exposure effect on the outcome variable at the population level, which eases interpretation of the results. In addition, propensity score methods mirror a randomized experiment because the study design is separated from the outcome analysis. In contrast, standard covariate adjustment approaches report the conditional treatment effect as an individual “*n*” unit increase of the outcome variable, change in Odds ratios or Hazard ratio and model specification is not separated from outcome analysis.

### Generation, differentiation, and treatment of hiPS-derived cardiomyocytes

Collection and subsequent reprogramming of peripheral blood mononuclear cells were performed as previously described [63]. Briefly, experiments were performed using iPS- derived CMs generated from iPSCs with passage numbers between 18 and 35. Differentiation into iPS-derived CMs was performed as previously described [63, 64]. Beating iPS- derived CMs were maintained in RPMI 1640 medium (Thermo Fisher Scientific) supplemented with B27 supplements (Thermo Fisher Scientific). All experiments were performed between day 31 and day 35 after initiation of differentiation. Human iPS-derived CMs were dissociated using prewarmed TrypLE select ×10 (Thermo Fisher Scientific) at 37 °C. Next, cells were detached, collected, and centrifuged using RPMI1640/B27 media. The cells were resuspended in RPMI1640/B27media supplemented with 10% knockout serum replacement (Thermo Fisher Scientific) and plated in Matrigel-coated six well dishes. After 24 h, media was changed to RPMI1640/B27 media. For immunostaining and RNA sequencing, hiPS CMs were exposed to doxorubicin 1 μM and/ or R-2HG (20 mM, (Sigma Aldrich, #H8378)) for 24 h in CDM3 medium.

### RNA sequencing and bioinformatics

To identify the effect of doxorubicin and R-2HG exposure on cardiomyocytes, RNA was isolated from cultured hiPS CMs using the RNeasy mini kit (Qiagen) following the instruction manual. Further analysis was performed at the Helmholtz Center for infection research in Braunschweig. The RNA sequencing library was generated by NEBNext Single Cell/Low Input RNA Library Prep Kit for Illumina according to the manufacturer’s protocols. The libraries were sequenced on a NovaSeq6000 sequencer PE50. Sequencing reads were aligned to human genome hg19 using the program STAR (v.2.5.2b) [65] with the help of Gencode v.25 [66] transcriptome annotation. Mapping to the transcriptome and gene expression quantification was done with Cufflinks [67] using Gencode v.25. Gene and transcript expression was quantified by FPKM (Fragments Per Kilobase per Million), TPM (Transcripts Per Million) values, as well as by fragment counts. Differentially regulated genes between R2-HG + DOX and DOX (or DOX and Control) with *p* < 0.05 were identified by R package “limma” based on log2(FPKM + 1) values. Gene ontology (GO, “Biological Process”) analysis was performed separately for up- and downregulated genes using the DAVID tool (v.6.8: https://david.ncifcrf.gov/). Heatmaps of differentially regulated genes were generated with the R package “ggplot”. The data discussed in this publication have been deposited in NCBI’s Gene Expression Omnibus and are accessible through GEO Series accession number GSE157282 (https://www.ncbi.nlm.nih.gov/geo/query/acc.cgi?acc=GSE157282).

### Assessment of sarcomere organization in hiPS-derived CMs

Immunostaining was performed as previously described [68]. Sarcomere organization was analyzed by confocal laser microscopy in hiPS-derived CMs immunostained for α-actinin (monoclonal anti-α-actinin (sarcomeric) antibody [Sigma Aldrich, #A7811)]. By using a semiquantitative grading system the sarcomere organization was classified. Ten percent of the cell area with sarcomere organization was defined as grade 1, 10–50% of the cell area with sarcomere organization as grade 2; 50–90% of the cell area with sarcomere organization grade 3, and >90% of the cell area with sarcomere organization as grade 4 [68].

### Statistical analysis

All data were analyzed using SPSS 24 for Windows (IBM SPSS statistics). All graphs were compiled with the use of Prism 7 software (GraphPad) or SPSS 24 for Windows (IBM SPSS statistics). Continuous variables are presented as median with range and means with standard deviations (SD). Analysis of data distribution was performed with the Kolmogorov–Smirnov and Shapiro–Wilk Test. Categorical variables are provided with absolute numbers (*n*) and percentages (%). We used the students *T*-test, one-way ANOVA or Mann Whitney *U* test (when appropriate) to compare continuous variables and the Pearson chi-square test to compare categorical variables. The null hypothesis was tested against a two-sided alternative hypothesis at a significance level of 5% [60]. As our study is the first study to start investigating whether the results from preclinical studies in mice might be also translated to patients, this exploratory study was designed to investigate primarily left ventricular ejection fraction as an outcome variable following inverse probability of treatment weighting. In a separate explorative analysis, overall survival endpoints, relapse free survival endpoints, and Hazard ratios were assessed in the unweighted (baseline) study cohort to characterize the study population and to report its comparability with previous studies. For exploratory purposes median follow-up time for survival was calculated according to the inverse Kaplan–Meier method. OS end points, measured from the date of diagnosis, were death (failure) and alive at last follow-up (censored). RFS end points, measured from the date of documented CR, were relapse (failure), death in CR (failure), and alive in CR at last follow-up (censored). For exploratory purposes the Kaplan–Meier method and log-rank tests were used to estimate the distribution of OS and RFS, and to compare differences between survival curves. The follow-up information was updated by means of clinic visits as well as telephone calls to patients, their doctors, or local registry offices.

## Supplementary information

Supplementary material
