# Supplementary material for: IDH1/2 mutations in acute myeloid leukemia patients and risk of coronary artery disease and cardiac dysfunction—a retrospective propensity score analysis
Source: Leukemia. 2020 Sep 18;35(5):1301–16. doi: 10.1038/s41375-020-01043-x (PMC8102189; doi:10.1038/s41375-020-01043-x)

IDH1/2 mutations in acute myeloid leukemia patients and risk of coronary artery disease and cardiac dysfunction - a retrospective propensity score analysis

Running Title: Cardiovascular risk in IDH-mutated AML

Badder Kattih[a,b,g,h], Amir Shirvani[a], Piroska Klement[e], Abel Martin Garrido[b], Razif Gabdoulline[e], Alessandro Liebich[e], Maximilian Brandes[e], Anuhar Chaturvedi[e], Timon Seeger[d,f], Felicitas Thol[e], Gudrun Göhring[c], Brigitte Schlegelberger[c], Robert Geffers[i], David John[g,h], Udo Bavendiek[a], Johann Bauersachs[a] , Arnold Ganser[e], Joerg Heineke[a, b, d, §,*] and Michael Heuser[e, #, *]

[a]Department of Cardiology and Angiology, Hannover Medical School, Carl-Neuberg Strasse 1, 30625 Hannover, Germany. [b]Department of Cardiovascular Physiology, European Center for Angioscience (ECAS), Medical Faculty Mannheim of Heidelberg University, Ludolf-Krehl-Strasse 7-11, 68167 Mannheim, Germany. [c]Department of Human Genetics, Hannover Medical School, Hannover, Germany. [d]German Center for Cardiovascular Research (DZHK), partner site Heidelberg/ Mannheim. [e]Department of Hematology, Hemostasis, Oncology and Stem Cell Transplantation, Hannover Medical School, Carl-Neuberg Strasse 1, 30625 Hannover, Germany. [f]Department of Medicine III, University Hospital Heidelberg; Im Neuenheimer Feld 410, 69120 Heidelberg. [g]Institute for Cardiovascular Regeneration, Goethe University Frankfurt, Frankfurt, Germany. [h]German Center for Cardiovascular Research (DZHK), Frankfurt, Germany. Theodor-Stern-Kai 7, 60590 Frankfurt, Germany). [i]Genome Analytics, Helmholtz Center for Infection Research, Braunschweig, Germany.

[§]Corresponding author: Department of Cardiovascular Physiology, European Center for Angioscience (ECAS), Medical Faculty Mannheim of Heidelberg University, Ludolf-Krehl Strasse 7-11, phone: +49 621-38371850, 68167 Mannheim, Germany. Email: Joerg.Heineke@medma.uni-heidelberg.de

#Corresponding author: Department of Hematology, Hemostasis, Oncology and Stem Cell Transplantation, Hannover Medical School, Carl-Neuberg Strasse 1, 30625, phone no.: +49 511 532 3720 Hannover, Germany. Email: heuser.michael@mh-hannover.de

*These authors contributed equally to this work

All authors declare no relevant conflicts of interest.

Table S1. Complete remission related to IDH mutational status in patients with AML

	unweighted cohort (total n=363)					
	***IDH* wildtype (total n=298)**	***IDH1* mutated (total n=26)**	***IDH2* mutated (total n=39)**	**P**	***IDH1/2* mutated (total n=65)**	**P**
**Complete remission (CR), (%)**	54.0 (161/298)	53.8 (14/26)	59.0 (23/39)	0.841	56.9 (37/65)	0.672
**CR (intensive induction chemotherapy), (%)**	65.7 (159/242)	63.6 (14/22)	74.2 (23/31)	0.615	69.8 (37/53)	0.566
**CR (without intensive induction Chemotherapy), (%)**	3.8 (2/52)	0.0 (0/4)	0.0 (0/8)	0.788	0.0 (0/12)	0.490

NOTE. Patients with acute promyelocytic leukemia were excluded from clinical correlations. Values are expressed as % (n/ total n).

Table S2. Overall survival and relapse-free survival of the AML study cohort

	IDH1/2 wildtype	*IDH1* mutated	*IDH2* mutated	*IDH1/2* mutated
2-year OS	49.9%	64.6%	59.8%	61.7%
5-year OS	36.2%	38.3%	38.7%	38.5%
2-year RFS	49.4%	54.2%	44.6%	48.2%
5-year RFS	37.1%	n/a	39.0%	44.8%

Abbreviation: OS= overall survival, RFS= relapse-free survival, n/a = not available.

Table S3. Baseline characteristics of the weighted AML study cohort (model1).

	weighted cohort (total n= 427)					
	**IDH wildtype (total n = 221)**	a.d. (n/ total n)	**IDH2 mutated (total n = 206)**	a.d. (n/ total n)	**p**	**SMD**
**Age, (years)**		(221/221)		(206/206)	0.836	0.012
median	61.0		64.0			
range	19.0-90.0		29.0 – 81.0			
**Age ≤60 years (young), (%)**	48.4	(107/221)	47.2	(97/206)	0.784	-0.019
**Age >60 years (old), (%)**	51.6	(114/221)	52.8	(109/206)		0.019
**Male sex, (%)**	57.8	(128/221)	61.3	(127/206)	0.436	
**AML history, (%)**					0.265	
De novo AML	68.3	(151/221)	67.2	(139/206)		-0.019
Secondary AML	23.4	(52/221)	28.0	(58/206)		0.086
Therapy-related AML	8.4	(18/221)	4.8	(10/206)		0.121
**WBC at diagnosis, (/µl)**		(218/221)		(200/206)	0.107	
median	9.6		13.9			
range	0.6-284.0		0.9-146.3			
**Platelet count at diagnosis, (/µl)**				(201/206)	0.006	
**median**	51.0		86.0			
**range**	2.0-523.0		7.0-979.0			
**Hemoglobin, (g/dl)**		(196/221)		(201/206)	0.004	
median	9.1		9.9			
range	4.7-16.0		5.5-16.6			
**Blood blasts, (%)**		(106/221)		(72/206)	0.003	
median	28.0		46.7			
range	0.0 -97.0		0.0-90.0			
**Bone marrow blasts, (%)**		(85/221)		(89/227)	0.011	
median	60.0		80.0			
range	0.0-99.0		10.0 – 92.0			
**Cytogenetic risk group, (%)**					0.026	
Favorable	21.1	(47/221)	11.4	(24/206)		-0.226
Intermediate	54.4	(120/221)	62.1	(128/206)		0.128
Adverse	24.5	(54/221)	26.5	(55/206)		0.038
**NPM1 mutation, (%)**	18.0	(40/221)	19.4	(40/206)	0.727	0.028
**FLT3-ITD presence, (%)**	17.6	(39/221)	14.5	(30/206)	0.375	-0.070
**FLT-TKD presence, (%)**	4.6	(10/221)	0.0	(0/206)	0.002	
**t(8;21), (%)**	3.5	(8/221)	4.5	(9/206)	0.685	
**inv(16), (%)**	2.0	(4/221)	4.8	(10/206)	0.078	
**MLL-MLLT3 t(9;11), (%)**	2.0	(4/221)	4.8	(10/206)	0.078	
**MLL partial tandem duplication, (%)**	4.2	(8/221)	4.8	(10/206)	0.074	
**ECOG performance status, (%)**					0.000	
ECOG 0-1	63.3	(140/221)	66.5	(137/206)		
ECOG 2-4	35.3	(78/221)	25.2	(52/206)		
**FAB subtype, (%)**					0.000	

M0	8.5	(14/221)	10.7	(15/206)		
M1	22.1	(35/221)	14.0	(20/206)		
M2	20.3	(32/221)	30.2	(43/206)		
M3	0.0	(0/221)	0.0	(0/206)		
M4	21.5	(34/221)	40.5	(58/206)		
M5	17.7	(28/221)	0.0	(0/206)		
M6	8.5	(14/221)	4.6	(7/206)		
M7	1.5	(2/221)	0.0	(0/206)		
**CMML, (%)**	1.5	(3/221)	3.1	(6/206)	0.264	
**Intensive induction therapy, (%)**	81.2	(180/221)	82.8	(171/206)	0.682	0.033
**Consolidation type, (%)**					0.647	
Chemotherapy	39.1	(74/221)	41.4	(80/206)		0.039
alloHCT	60.9	(115/221)	58.6	(113/206)		-0.039
**Extramedullary AML, (%)**	8.1	(18/221)	6.2	(13/206)	0.457	
**Cardiovascular characteristics**						
**CAD, (%)**	5.0	(11/221)	4.8	(10/206)	0.953	-0.005
**Valvular disease, (%)**	1.6	(3/221)	1.2	(2/206)	0.625	-0.015
**Heart failure, (%)**	0.0	(0/221)	0.0	(0/206)		0.000
**Cardiovascular Risk**					0.519	
0-1 risk factors	96.6	(214/221)	95.2	(196/206)		-0.057
2-4 risk factors	3.4	(8/221)	4.8	(10/206)		0.057
**Sinusrhythm, (%)**	94.6	(209/221)	95.2	(196/206)	0.788	0.021
**Systolic blood pressure, (mmHg)**		(88/221)		(110/206)	0.207	
median	130.0		130.0			
range	80.0-180.0		110.0 – 150.0			
**Diastolic blood pressure, (mmHg)**		(87/221)		(110/206)	0.165	
median	75.0		80.0			
range	40.0-105.0		70.0-80.0			
**Heart rate, (beat per min)**		(128/221)		(127/206)	0.271	
median	88.0		84.0			
range	60.0-152.0		66.0 – 108.0			
**ACEi/ARB, (%)**	17.6	(39/221)	17.0	(35/206)	0.858	-0.014
**ß-blocker, (%)**	17.0	(38/221)	14.0	(29/226)	0.376	-0.068
**MR-antagnoists, (%)**	0.0	(0/221)	0.0	(0/206)		0.000

Values are expressed as median and range or % (n/ total n). a.d.= available data. Abbreviation: alloHCT= allogeneic hematopoietic cell transplantation, CMML= chronic myelomonocytic leukemia, ECOG= Eastern Co-operative Oncology Group, FLT3-ITD= FMS-like tyrosine kinase 3 internal tandem duplication, NPM1= Nucleophosmin 1, SMD= standardized mean difference, SMD provided for baseline characteristics included in the propensity score model (model1).

Table S4. Baseline characteristics of the weighted AML study cohort (model2).

	weighted cohort (total n= 434)					
	**IDH1/2 wildtype (total n = 216)**	a.d. (n/ total n)	**pooled IDH1/2 mutated (total n = 218)**	a.d. (n/ total n)	**p**	**SMD**
**Age, (years)**		(216/216)		(218/218)	0.388	0.069
median	58.5		63.0			
range	19.0-90.0		29.0 – 82.0			
**Age ≤60 years (young), (%)**	45.5	(118/216)	47.9	(104/218)	0.626	0.034
**Age >60 years (old), (%)**	54.5	(98/216)	52.1	(114/218)		-0.034
**Male sex, (%)**	57.1	(123/216)	61.7	(135/218)	0.291	
**AML history, (%)**					0.030	
De novo AML	71.1	(154/216)	67.6	(147/218)		
Secondary AML	19.6	(42/216)	27.8	(61/218)		
Therapy-related AML	9.3	(20/216)	4.6	(10/218)		
**WBC at diagnosis, (/µl)**		(216/216)		(218/218)	0.394	0.041
median	9.9		8.4			
range	0.6-284.0		0.7-206.1			
**Platelet count at diagnosis, (/µl)**		(216/216)		(218/218)	0.552	0.059
median	64.0		74.0			
range	2.0-523.0		7.0-979.0			
**Hemoglobin, (g/dl)**		(210/216)		(218/218)	0.510	
median	9.1		9.3			
range	4.7-16.0		5.4-16.6			
**Blood blasts, (%)**		(109/216)		(218/218)	0.000	
median	26.0		46.7			
range	0.0 -95.0		0.0-90.0			
**Bone marrow blasts, (%)**		(90/232)		(90/218)	0.029	
median	50.0		80.0			
range	0.0-90.0		10.0 – 96.0			
**Cytogenetic risk group, (%)**					0.229	
Favorable	22.2	(39/216)	22.6	(49/218)		0.058
Intermediate	57.8	(125/216)	49.7	(108/218)		-0.125
Adverse	24.0	(52/216)	27.7	(60/218)		0.087
**NPM1 mutation, (%)**	17.8	(38/216)	19.0	(41/218)	0.743	0.008
**FLT3-ITD presence, (%)**	17.7	(38/216)	19.2	(42/218)	0.653	0.032
**FLT-TKD presence, (%)**	4.7	(10/216)	0.0	(0/218)	0.001	
**t(8;21), (%)**	3.9	(8/216)	1.2	(3/218)	0.123	
**inv(16), (%)**	2.8	(6/216)	4.6	(10/218)	0.317	
**MLL-MLLT3 t(9;11), (%)**	1.5	(3/216)	7.0	(15/218)	0.004	
**MLL partial tandem duplication, (%)**	4.3	(8/216)	9.6	(19/218)	0.037	
**ECOG performance status, (%)**					0.000	
Grade 0-1	65.7	(142/216)	67.0	(146/218)		
Grade 2-4	34.3	(74/216)	33.0	(72/218)		

**FAB subtype, (%)**					0.002	
M0	7.7	(12/216)	12.2	(17/218)		
M1	25.1	(40/216)	17.5	(25/218)		
M2	21.1	(34/216)	25.0	(35/218)		
M3	0.0	(0/232)	0.0	(0/218)		
M4	20.3	(32/216)	33.8	(48/218)		
M5	17.8	(28/232)	10.7	(15/218)		
M6	6.1	(10/232)	0.8	(1/218)		
M7	1.8	(3/216)	0.0	(0/218)		
**CMML, (%)**	1.8	(4/216)	18.9	(41/218)	0.006	
**Intense therapy, (%)**	80.6	(174/232)	81.1	(177/218)	0.866	0.017
**Consolidation type, (%)**					0.799	
Chemotherapy	39.2	(72/216)	40.4	(80/218)		
alloHCT	60.8	(112/216)	59.6	(118/218)		
**Extramedullary AML, (%)**	8.9	(19/216)	10.6	(23/218)	0.397	
**Cardiovascular characteristics**						
**CAD, (%)**	8.1	(19/216)	9.5	(21/218)	0.516	0.030
**Valvular disease, (%)**	7.7	(17/216)	2.8	(6/218)	0.136	-0.005
**Heart failure, (%)**	0.0	(0/216)	0.0	(0/218)		0.000
**Cardiovascular Risk, (%)**					0.478	
0-1 risk factors	90.3	(195/216)	92.4	(201/218)		0.091
2-4 risk factors	9.7	(21/216)	7.6	(17/218)		-0.091
**Sinusrhythm, (%)**	92.9	(201/216)	92.0	(200/218)	0.606	0.068
**Systolic blood pressure, (mmHg)**		(90/216)		(105/218)	0.017	
median	130.0		120.0			
range	90.0-180.0		110.0 – 150.0			
**Diastolic blood pressure, (mmHg)**		(89/216)		(105/218)	0.267	
median	75.0		80.0			
range	50.0-105.0		70.0-80.0			
**Heart rate, (beats per min)**		(129/216)		119/218	0.276	
median	87.0		88.0			
range	60.0-152.0		66.0 – 112.0			
**ACEi/ARB, (%)**	20.0	(43/216)	19.8	(43/218)	0.962	-0.011
**ß-blocker, (%)**	18.1	(39/216)	17.6	(38/218)	0.865	-0.028
**MR-antagnoists, (%)**	0.0	(0/216)	0.0	(0/218)	1.000	-0.132

Values are expressed as median and range or % (n/ total n). a.d.= available data. Abbreviation: alloHCT= allogeneic hematopoietic cell transplantation, CMML= chronic myelomonocytic leukemia, ECOG= Eastern Co-operative Oncology Group, FLT3-ITD= FMS-like tyrosine kinase 3 internal tandem duplication, NPM1= Nucleophosmin 1, SMD= standardized mean difference, SMD provided for baseline characteristics included in the propensity score model (model2).

Table S5. Echocardiographic outcomes related to *IDH* mutational status in patients with AML after IPW (*IDH1/2* wildtype vs. *IDH2* mutated).

unweighted cohort (total n= 337)

	***IDH1/2* wildtype** (total n = 298)	***IDH2* mutated** (total n = 39)	***p** (WT vs. Mut)	**#p** (WT t0 vs tx)	**#p** (Mut t0 vs tx)	**ATE (95%CI)** (absolute difference LVEF)
**Echocardiography pre-AML therapy (t_0)**						
Ejection fraction (%)	58.6.0 ±8.0 (71/298)	57.6 ±4.5 (8/39)	0.770			-1.0 % (-9.58 to 7.58)
**Echocardiography during AML therapy (t_1)**						
Ejection fraction (%)	55.0 ±9.3 (74/298)	57.6 ±4.9 (7/39)	0.474	0.018	0.999	2.6 % (-6.49 to 11.69)
**Echocardiography during AML therapy (t_2)**						
Ejection fraction (%)	54.8 ±9.7 (43/298)	51.2±15.5 (5/39)	0.406	0.032	0.258	-3.6 % (-14.47 to 7.27)
**Echocardiography during AML therapy (t_3)**						
Ejection fraction (%)	53.4±10.5 (22/298)	44.6±12.3 (7/39)	0.028	0.020	0.016	-8.8 % (-18.78 to 1.18)

Timepoints ($t_{0\text{-}3}$) are calculated as tmean and median time (months) from date of diagnosis to date of echocardiography. t_0 (median:0 months, range -10 to 2, mean 0, SD ±2 (52/52), t_1 (median:3 months, range 0 to 53, mean 6, SD ±9 (75/75), t_2 (median:9 months, range 1 to 54 mean 13, SD ±13 (34/34), t_3 (median:15 months, range 2 to 161, mean 23, SD ±31 (19/19). Values are expressed as means and ±standard deviation. (n/ total n) denotes available data. *comparing wildtype *IDH* with mutated *IDH1* at the corresponding timepoint (t_x), # comparing the corresponding timepoint within the same group with timepoint t_0. t_0 denotes the timepoint before AML therapy and $t_{1\text{-}3}$ show the ejection fraction at different timepoints during AML therapy.Mut, mutated; WT, wildtype.

weighted cohort (total n=418)

	***IDH1/2* wildtype** (total n = 221)	***IDH2* mutated** (total n = 197)	***p** (WT vs. Mut)	**#p** (WT t0 vs tx)	**#p** (Mut t0 vs tx)	**ATE (95%CI)** (absolute difference LVEF)
**Echocardiography pre-AML therapy (t_0)**						
Ejection fraction (%)	59.0 ±8.1 (53/221)	55.1 ±4.2 (34/197)	0.053			-3.9 % (-8.92 to 1.12)
**Echocardiography post-AML therapy (t_1)**						
Ejection fraction (%)	54.9 ±10.2 (63/221)	56.2 ±3.7 (42/197)	0.475	0.025	0.562	1.3 % (-3.25 to 5.85)
**Echocardiography post-AML therapy (t_2)**						
Ejection fraction (%)	54.8 ±10.8 (36/221)	48.2 ±13.7 (27/197)	0.005	0.046	0.002	-6.6 % (-12.42 to -0.78
**Echocardiography post-AML therapy (t_3)**						
Ejection fraction (%)	54.6±9.8 (21/221)	46.3±10.7 (16/197)	0.005	0.080	0.001	-8.3 % (-15.88 to -0.7159)

Timepoints ($t_{0\text{-}3}$) are calculated as the median time (months) from date of diagnosis to date of echocardiography. t_0 (median:0 months, range -10 to 1, mean 0, SD ±2 (87/87), t_1 (median:3 months, range 0 to 48, mean 5, SD ±7 (105/105), t_2 (median:9 months, range 1 to 53, mean 5, SD ±16 (63/63), t_3 (median:11 months, range 2 to 162, mean 25, SD ±32 (37/37). Values are expressed as means and ±standard deviation. (n/ total n) denotes available data. *comparing wildtype *IDH* with mutated *IDH1* at the corresponding timepoint (t_x), # comparing the corresponding timepoint within the same group with timepoint t0. t0 denotes the timepoint before AML therapy and t1-3 show the ejection fraction at different timepoints during AML therapy.Mut, mutated; WT, wildtype.

Table S6. Echocardiographic outcomes related to *IDH* mutational status in AML after IPW (*IDH1/2* wildtype vs. pooled *IDH1/2* mutated).

unweighted cohort (total n= 363)

	***IDH1/2* wildtype** (total n = 298)	***IDH1/2* mutated** (total n = 65)	***p** (WT vs. Mut)	**#p** (WT t_0 vs t_x)	**#p** (Mut t_0 vs t_x)	**ATE (95%CI)** (absolute difference LVEF)
**Echocardiography pre-AML therapy (t_0)**						
Ejection fraction (%)	58.6.0 ±8.0 (71/298)	57.6 ±4.5 (8/65)	0.710			1.0 % (-5.74 to 7.74)
**Echocardiography during AML therapy (t_1)**						
Ejection fraction (%)	55.0 ±9.3 (74/298)	57.6 ±4.9 (7/65)	0.398	0.018	0.999	2.2 % (-4.33 to 8.73)
**Echocardiography during AML therapy (t_2)**						
Ejection fraction (%)	54.8 ±9.7 (43/298)	51.2±15.5 (5/65)	0.017	0.032	0.258	-8.5 % (-17.37 to -0.373)
**Echocardiography during AML therapy (t_3)**						
Ejection fraction (%)	53.4±10.5 (22/298)	44.6±12.3 (7/65)	0.028	0.020	0.016	-8.8 % (-18.80 to 1.2)

Timepoints (t_{0-3}) are calculated as mean and median time (months) from date of diagnosis to date of echocardiography. t_0 (median:0 months, range -10 to 2, mean 0, SD±2 (83/85), t_1 (median:3 months, range 0 to 53, mean 6, SD±9 (88/89), t_2 (median:9 months, range 1 to 54, mean 13, SD±13 (51/51), t_3 (median:5 months, range 2 to 161, mean 23, SD±31 (29/29).Values are expressed as means and ±standard deviation. (n/ total n) denotes available data. *comparing wildtype *IDH* with mutated *IDH1* at the corresponding timepoint (t_x), # comparing the corresponding timepoint within the same group with timepoint t_0. t_0 denotes the timepoint before AML therapy and t_{1-3} show the ejection fraction at different timepoints during AML therapy.Mut, mutated; WT, wildtype.

weighted cohort (total n=434)

	***IDH1/2* wildtype** (total n = 216)	***IDH1/2* mutated** (total n = 218)	***p** (WT vs. Mut)	**#p** (WT t_0 vs t_x)	**#p** (Mut t_0 vs t_x)	**ATE (95%CI)** (absolute difference LVEF)
**Echocardiography pre-AML therapy (t_0)**						
Ejection fraction (%)	58.5 ±8.7 (53/216)	59.2 ±7.0 (42/218)	0.7305			0.7 % (-4.39 to 5.79)
**Echocardiography during AML therapy (t_1)**						
Ejection fraction (%)	54.6 ±10.9 (61/216)	55.8 ±8.4 (58/218)	0.506	0.043	0.078	1.2 % (-3.32 to 5.72)
**Echocardiography during AML therapy (t_2)**						
Ejection fraction (%)	54.4 ±10.9 (35/216)	45.2 ±9.5 (31/218)	0.000	0.066	0.000	-9.4 % (-20.94 to -6.06)
**Echocardiography during AML therapy (t_3)**						
Ejection fraction (%)	55.4±10.1 (18/216)	41.9±13.8 (28/218)	0.000	0.265	0.000	-13.5 % (-20.94 to -6.06)

Timepoints (t_{0-3}) are calculated as mean and median time (months) from date of diagnosis to date of echocardiography. t_0 (median:0 months, range -10 to 1, mean 0, SD±2 (94/95), t_1 (median:3 months, range 0 to 53, mean 5, SD±7 (119/119), t_2 (median:6 months, range 1 to 54, mean 9, SD±9 (66/66), t_3 (median:6 months, range 2 to 41, mean 10, SD±9 (45/45).

Values are expressed as means and ±standard deviation. (n/ total n) denotes available data. *comparing wildtype *IDH* with mutated *IDH1* at the corresponding timepoint (t_x), # comparing the corresponding timepoint within the same group with timepoint t_0. t_0 denotes the timepoint before AML therapy and t_{1-3} show the ejection fraction at different timepoints during AML therapy.Mut, mutated; WT, wildtype.

Table S7. Echocardiographic outcomes related to *IDH* mutational status in patients with AML after IPW (*IDH* wildtype vs. *IDH1* mutated).

	unweighted cohort (total n= 324)					
	***IDH1/2* wildtype (total n = 298)**	***IDH1* mutated (total n = 26)**	***p (WT vs. Mut)**	**#p (WT t_0 vs t_x)**	**#p-value (Mut t_0 vs t_x)**	**ATE (95%CI) (absolute difference LVEF)**
**Echocardiography pre-AML therapy (t_0)**						
Ejection fraction (%)	58.6.0 ±8.0 (71/298)	62.1 ±5.2 (6/26)	0.0370			3.5 % (-4.18 to 11.18)
**Echocardiography during AML therapy (t_1)**						
Ejection fraction (%)	55.0 ±9.3 (74/298)	56.9 ±11.3 (8/26)	0.578	0.018	0.349	1.9 % (-4.82 to 8.62)
**Echocardiography during AML therapy (t_2)**						
Ejection fraction (%)	54.8 ±9.7 (43/298)	38.0±7.5 (3/26)	0.002	0.032	0.004	-16.8 % (-27.59 to -6.01)
**Echocardiography during AML therapy (t_3)**						
Ejection fraction (%)	53.4±10.5 (22/298)	39.0±15.0 (3/26)	0.011	0.020	0.005	-14.4 % (-25.52 to -3.28)

Timepoints (t_{0-3}) are calculated as mean and median time (month) from date of diagnosis to date of echocardiography. t_0 (median:0 month, range -10 to 2, mean 0, SD±2 (75/77), t_1 (median:2 month, range 0 to 53, mean 6, SD±9 (81/82), t_2 (median:9 month, range 1 to 54, mean 12, SD±12 (46/46), t_3 (median:14 month, range 2 to 161, mean 24, SD±32 (25/25). Values are expressed as means and ±standard deviation. (n/ total n) denotes available data. *comparing wildtype *IDH* with mutated *IDH1* at the corresponding timepoint (t_x), # comparing the corresponding timepoint within the same group with timepoint t_0. t_0 denotes the timepoint before AML therapy and t_{1-3} show the ejection fraction at different timepoints during AML therapy.Mut, mutated; WT, wildtype.

Table S8. Echocardiographic timing related to IDH mutational status in patients with AML

	unweighted cohort		
	***IDH1/2* wiltype**	***IDH2* mutated**	**Total cohort**
**Timepoint t0 (months)**	Mean 0 (SD±2)<br>Median 0 (range -10 to 2)	Median 0 (range 0 to 1)<br>Mean 0 (SD±0)	Median 0 (range -10 to 2)<br>Mean 0 (SD±2)
**Timepoint t1 (months)**	Mean 6 (SD±9)<br>Median 3 (range 0 to 53)	Mean 5 (SD±4)<br>Median 3 (range 1 to 10)	Mean 6 (SD±9)<br>Median 3 (range 0 to 53)
**Timepoint t2 (months)**	Mean 13 (SD±12)<br>Median 9 (range 1 to 54)	Mean 17 (SD±20)<br>Median 12 (range 3 to 53)	Mean 13 (SD±13)<br>Median 9 (range 1 to 54)
**Timepoint t3 (months)**	Mean 23 (SD±33)<br>Median 15 (range 2 to 161)	Mean 22 (SD±25)<br>Median 14 (range 3 to 57)	Mean 23 (SD±31)<br>Median 15 (range 2 to 161)

	unweighted cohort		
	***IDH1/2* wiltype**	***IDH1* mutated**	**Total cohort**
**Timepoint t0 (months)**	Mean 0 (SD±2)<br>Median 0 (range -10 to 2)	Mean 0 (SD±0)<br>Median 0 (range 0 to 1)	Mean 0 (SD±2)<br>Median 0 (range -10 to 2)
**Timepoint t1 (months)**	Mean 6 (SD±9)<br>Median 3 (range 0 to 53)	Mean 3 (SD±4)<br>Median 2 (range 0 to 11)	Mean 6 (SD±9)<br>Median 2 (range 0 to 53)
**Timepoint t2 (months)**	Mean 13 (SD±12)<br>Median 9 (range 1 to 54)	Mean 6 (SD±6)<br>Median 3 (range 1 to 13)	Mean 12 (SD±12)<br>Median 9 (range 1 to 54)
**Timepoint t3 (months)**	Mean 23 (SD±33)<br>Median 15 (range 2 to 161)	Mean 25 (SD±27)<br>Median 14 (range 6 to 56)	Mean 24 (SD±32)<br>Median 14 (range 2 to 161)

	unweighted cohort		
	***IDH1/2* wiltype**	***IDH1/2* mutated**	**Total cohort**
**Timepoint t0 (months)**	Mean 0 (SD±2)<br>Median 0 (range -10 to 2)	Mean 0 (SD±0)<br>Median 0 (range 0 to 1)	Mean 0 (SD±1)<br>Median 0 (range -10 to 2)
**Timepoint t1 (months)**	Mean 6 (SD±9)<br>Median 3 (range 0 to 53)	Mean 3 (SD±4)<br>Median 2 (range 0 to 11)	Mean 6 (SD±9)<br>Median 2 (range 0 to 53)
**Timepoint t2 (months)**	Mean 13 (SD±12)<br>Median 9 (range 1 to 54)	Mean 6 (SD±6)<br>Median 3 (range 1 to 13)	Mean 12 (SD±12)<br>Median 9 (range 1 to 54)
**Timepoint t3 (months)**	Mean 23 (SD±33)<br>Median 15 (range 2 to 161)	Mean 25 (SD±27)<br>Median 14 (range 6 to 56)	Mean 24 (SD±32)<br>Median 14 (range 2 to 161)

	weighted cohort		
	***IDH1/2* wiltype**	***IDH1/2* mutated**	**Total cohort**
**Timepoint t0 (months)**	Mean 0 (SD±2)<br>Median 0 (range -10 to 1)	Mean 0 (SD±0)<br>Median 0 (range 0 to 1)	Mean 0 (SD±2)<br>Median 0 (range -10 to 1)
**Timepoint t1 (months)**	Mean 6 (SD±8)<br>Median 3 (range 0 to 53)	Mean 4 (SD±4)<br>Median 3 (range 0 to 11)	Mean 5 (SD±7)<br>Median 3 (range 0 to 53)
**Timepoint t2 (months)**	Mean 11 (SD±11)<br>Median 6 (range 1 to 54)	Median 6 (range 1 to 13)<br>Mean 7 (SD±5)	Median 6 (range 1 to 54)<br>Mean 9 (SD±9)
**Timepoint t3 (months)**	Mean 16 (SD±10)<br>Median 14 (range 5 to 41)	Mean 6 (SD±5)<br>Median 5 (range 2 to 16)	Mean 10 (SD±9)<br>Median 6 (range 2 to 41)

	weighted cohort		
	***IDH*1/2 wildtype**	***IDH2* mutated**	**Total cohort**
**Timepoint t0 (months)**	Mean 0 (SD±2)<br>Median 0 (range -10 to 1)	Mean 0 (SD±0)<br>Median 0 (range 0 to 1)	Mean 0 (SD±2)<br>Median 0 (range -10 to 1)
**Timepoint t1 (months)**	Mean 5 (SD±8)<br>Median 3 (range 0 to 48)	Mean 5 (SD±3)<br>Median 3 (range 0 to 10)	Mean 5 (SD±7)<br>Median 3 (range 0 to 48)
**Timepoint t2 (months)**	Mean 12 (SD±12)<br>Median 7 (range 1 to 49)	Mean 20 (SD±20)<br>Median 12 (range 6 to 53)	Mean 15 (SD±16)<br>Median 9 (range 1 to 53)
**Timepoint t3 (months)**	Mean 25 (SD±36)<br>Median 15 (range 2 to 161)	Mean 25 (SD±27)<br>Median 3 (range 3 to 57)	Mean 25 (SD±32)<br>Median 11 (range 2 to 161)

Timepoints ($t_{0\text{-}3}$) are calculated as the mean and median time (months) from date of diagnosis to date of echocardiography. t0 denotes the timepoint before AML therapy and t1-3 show the ejection fraction at different timepoints during AML therapy.

Mut, mutated; WT, wildtype.

Supplemental Figure S1

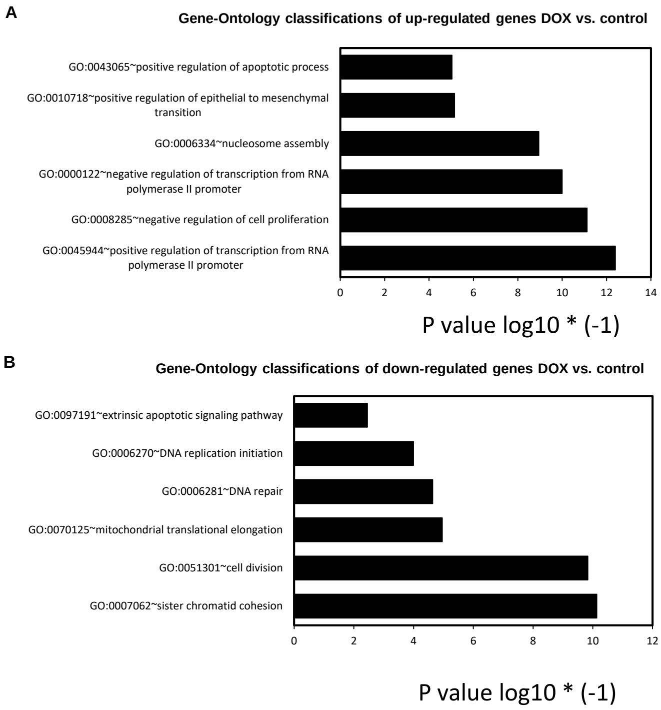

Supplement: Supplementary file 1 — Supplementary material [file 41375_2020_1043_MOESM1_ESM.pdf]
